# Habitat suitability mapping of *Anopheles darlingi *in the surroundings of the Manso hydropower plant reservoir, Mato Grosso, Central Brazil

**DOI:** 10.1186/1476-072X-6-7

**Published:** 2007-03-07

**Authors:** Peter Zeilhofer, Emerson Soares dos Santos, Ana LM Ribeiro, Rosina D Miyazaki, Marina Atanaka dos Santos

**Affiliations:** 1 Department of Geography, Federal University of Mato Grosso, Av. F. Corrêa, Cuiabá, Brazil; 2 Institute of Biology, Federal University of Mato Grosso, Av. F. Corrêa, Cuiabá, Brazil; 3 Institute of Public Health, Federal University of Mato Grosso, Av. F. Corrêa, Cuiabá, Brazil

## Abstract

**Background:**

Hydropower plants provide more than 78 % of Brazil's electricity generation, but the country's reservoirs are potential new habitats for main vectors of malaria. In a case study in the surroundings of the Manso hydropower plant in Mato Grosso state, Central Brazil, habitat suitability of *Anopheles darlingi *was studied. Habitat profile was characterized by collecting environmental data. Remote sensing and GIS techniques were applied to extract additional spatial layers of land use, distance maps, and relief characteristics for spatial model building.

**Results:**

Logistic regression analysis and ROC curves indicate significant relationships between the environment and presence of *An. darlingi*. Probabilities of presence strongly vary as a function of land cover and distance from the lake shoreline. Vector presence was associated with spatial proximity to reservoir and semi-deciduous forests followed by *Cerrado *woodland. Vector absence was associated with open vegetation formations such as grasslands and agricultural areas. We suppose that non-significant differences of vector incidences between rainy and dry seasons are associated with the availability of anthropogenic breeding habitat of the reservoir throughout the year.

**Conclusion:**

Satellite image classification and multitemporal shoreline simulations through DEM-based GIS-analyses consist in a valuable tool for spatial modeling of *A. darlingi *habitats in the studied hydropower reservoir area. Vector presence is significantly increased in forested areas near reservoirs in bays protected from wind and wave action. Construction of new reservoirs under the tropical, sub-humid climatic conditions should therefore be accompanied by entomologic studies to predict the risk of malaria epidemics.

## Background

Malaria is caused by protozoan parasites of the genus Plasmodium and is transmitted, in Central Brazil, principally by *Anopheles darlingi *Root and four other dipters species of the Anophelines genus: *Anopheles aquasalis*, *Anopheles albitarsis*, *Anopheles cruzi *and *Anopheles bellator*. *An. darlingi *is considered the most anthropophilic and endophilic species among the Amazonian anophelines [1]. The preferential reproduction habitat of *A. darlingi *is in areas of still, clean water, and adults can fly up to 7 km for resting and feeding [2].

In Brazil, malaria is endemic in the Central Amazon region. Roberts et al. [3] reported an increasing incidence of malaria in the Amazon in the early and mid-nineties due to changes in strategies for malaria control (reduction of residual house-spraying). By 2002, cases had reduced slightly, to 349,873 cases [4]. More than 99% of all cases in Brazil have been observed in the Amazon region, which includes Mato Grosso state [5]. In southern Mato Grosso, *An. darlingi *is abundant, but no malaria epidemics have yet been observed. Epidemic outbreaks, however, are a latent risk, particularly in areas with a high density of vectors.

Hydropower plants provide more than 78 % of Brazil's electricity generation. With an estimated growth in demand of about 4.5 % per year, implantation of new power plants is ongoing [6]. The filling phase of the Manso hydropower plant reservoir, located about 100 km north of Cuiabá, the capital of Mato Grosso state, was initiated in November 1998 and concluded in 2001. At its highest operational levels, the reservoir covers an area of about 427 km^2^, and is a potential new habitat for malaria vectors [7].

Remote Sensing and Geographical Information Systems (GIS) has emerged as an innovative and important component in public health and epidemiology [8], and has been widely used for monitoring, surveillance and mapping of vector habitat and spatial modeling of vector-borne diseases [9]. Thomson et al. [10], as well as Rogers et al. [11], have given overviews of applicability of Earth-observation satellites for the study of ecology and forecast of malaria. Pope et al. [12], Beck et al. [13] and Roberts et al. [14] have given application examples for the mapping of malaria vectors, pointing out the suitability of high resolution Remote Sensing (RS) data, such as from the Landsat ETM or SPOT systems, for vegetation and land cover mapping. Newly available imagery of very high-resolution RS systems such as Ikonos and Quickbird were found to improve mapping results of small larval habitats or vegetation cover in highly structured landscapes [15,16].

In their comprehensive overview of applicability of RS techniques for vector disease analysis, Beck et al. [9] mentioned factors such as vegetation/crop type, vegetation green up, deforestation and landscape structure as relevant for the evaluation of malaria breeding, resting and feeding habitats. Malaria transmission in the Brazilian Amazon has been found to be positively related to deforestation [17], due to the increase of populations which have direct contact with vectors and which are commonly living in precarious conditions of habitation, nutrition and health care, factors favoring malaria transmission and complicating vector control [18,19]. Vittor et al. in 2006 [20] reported a striking increase in human biting rates of *An. darlingi *densities in deforested areas in the Peruvian Amazon. Castro et al. [21] mentioned for a study area in Rondonia that malaria transmission in early stages of frontier settlement is dominated by environmental risk, while in consolidated occupations infection risk is mainly determined by behavioral factors.

Singer & Castro [22] pointed out that principal natural breeding places of *An. darlingi *in the Amazon are at the forest margins; when inside undisturbed forests, ideal breeding habitats are rare, since standing water is acidic and the partial shade favored by this species is absent. In addition, the construction of rural roads frequently creates permanent breeding sites for *An. darlingi*, as a consequence of poor drainage [21]. Studies in the savannah ecosystems of the Brazilian Amazon are still rare, as they are not considered to be endemic regions [23].

The monitoring of wetlands – both natural and artificial – and flooding is fundamental, as bodies of water are the breeding habitat of *Anopheles *larva [2]. In Belize, *An. darlingi *densities have been found to be positively related to riverine vegetation types [24]. Principal reproduction habitats were shaded or partly shaded patches of floating debris and submerged plants along creek and river margins. The comprehensive study of Keiser et al. [25] conclude that the implantation of dams favor habitat suitability of malaria vectors. In Brazil, increase of malaria transmission has been reported for the great reservoirs of Balbina, Tucuriu, Samuel and Itaipu [25,26]. Vasconcelos et al [17], as well as Tadei et al [27], mentioned that the accumulation of nutrients in reservoirs can favor growth of aquatic vegetation, important reproduction habitats for *An darlingi*. As shown for the Lower Kihansi Hydropower plant in Tanzania, artificial bodies of water can even introduce malaria into areas not known to have the disease [28]. On a local scale, the expectation of an increase of vector densities around reservoirs is supposed to vary as a function of distance from the shoreline and the proximity to portions of the reservoir suitable as breeding habitats [17,25,29]. In GIS-based approaches these factors can be derived from Digital Elevation Model (DEM) analysis [15].

Mosquito activity is supposed to be strongly influenced by environmental factors such as temperature, humidity, wind speed and moon phase and should therefore be considered in spatial model building [23,30,31].

**Figure 1:**
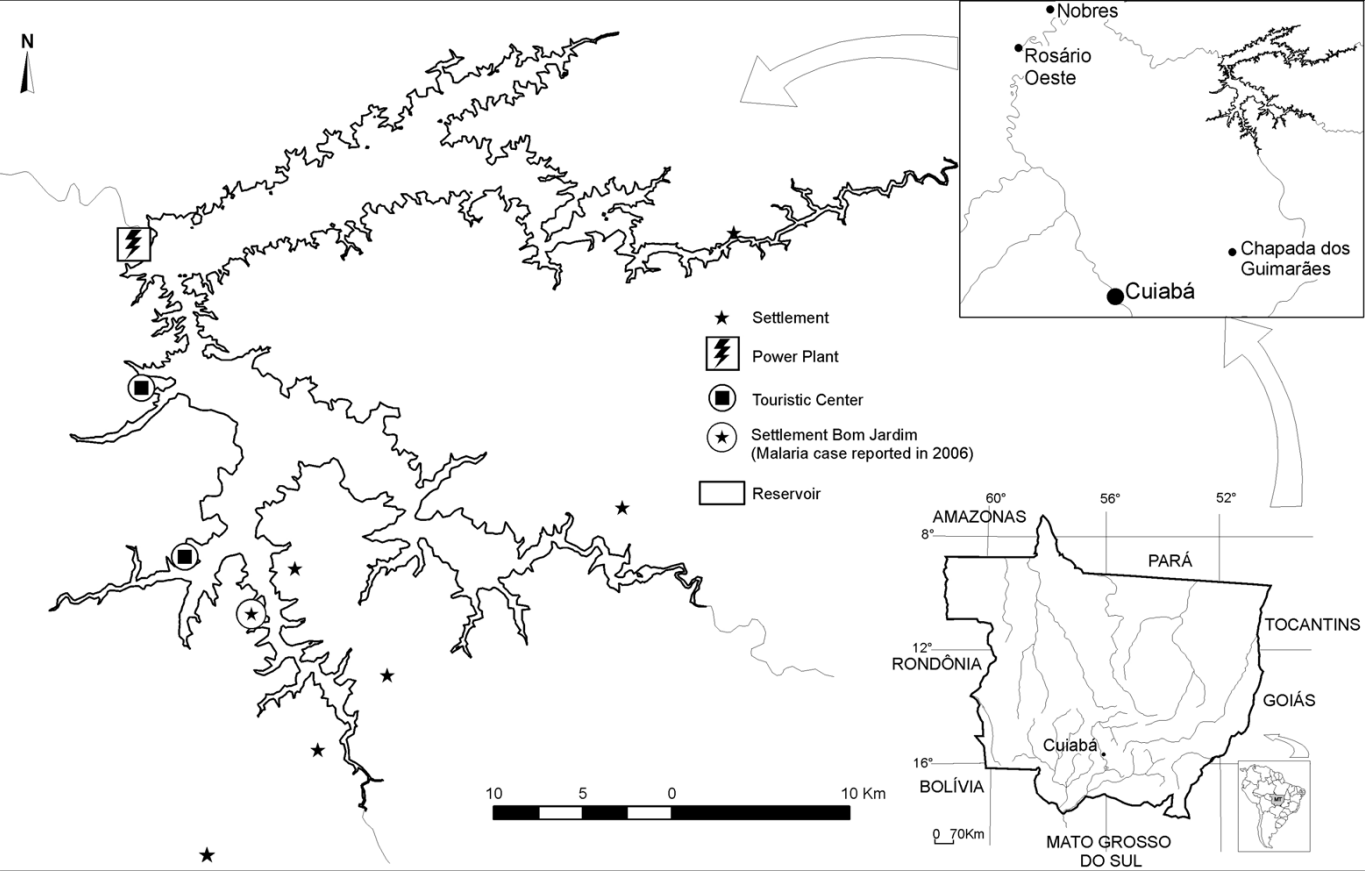
Study area.

In the present study we report on our experience of applying satellite-based remote sensing of vegetation and land-use cover and geographic information system (GIS) analytic techniques based on DEM to the study of habitat suitability of the malaria vector *Anopheles darlingi *Root. In our study area, the Manso hydropower plant influence area in the Central south of Mato Grossso state (Central Brazil), malaria is not endemic, but in 2006, a case was reported for the "Bom Jardim" settlement (Fig. 1). Despite the resettled former inhabitants of the areas flooded by the reservoir, the study area is frequently visited by tourists, mainly from the city of Cuiabá, which use the lake for recreation purposes on weekends. As monkey fauna, which is believed to be the main animal host of malaria parasites, is common in the Cerrado woodlands and gallery forests [32], presented habitat evaluation should be considered a potential transmission risk assessment. Our focus is on the influence of the Manso hydropower plant reservoir on vector incidence, applying logistic regressions and testing model sensitivity and specificity by ROC curves. Spatial and temporal distribution of the vector is related to reservoir climatic and environmental factors (temperature, humidity, rainfall, land cover, distance to potential reproduction habitat, reservoir shoreline morphology) in order to present a detailed map of vector habitat suitability which could provide the basis for the development of a malaria early warning system.

## Methods

### Study area

Figure 1 shows the location of the Manso powerplant reservoir at 15°11'25" and 14°44'42" southern latitude, and 55°52'46" and 55°19'23" western length, about 100 km to the north of Cuiabá, in the southern part of the Brazilian state of Mato Grosso. At its maximum operation level of 287.5 m NN, the reservoir covers an area of approximately 427 km^2^. Average annual temperature of the semi humid tropical climate is about 26° degrees Celsius, with an estimated precipitation height of about 1,750 mm with two distinct periods: about 80% of precipitation occurs between November and April, while mean monthly precipitation in June through August is above 20 mm. The study area is located in the Central Brazilian Cerrado region, a savannah biome, which, principally due to edaphic factors, includes formations ranging from open grasslands to dense woodlands. Vegetation classification schemes distinguish two formations of denser scrub- or woodlands in the Cerrado biome: the Cerrado strictu sensu (dense, mostly mesophyllous shrubs with a grass understory) and Cerradão (woodland, mostly mesophyllous trees) [33]. The term savannah used here refers to these two vegetation formations. Native woodland and scrublands surrounding the reservoir have been partially deforested and used for extensive cattle farming. Semi-deciduous forests are developed along the hydrographical network and at the ramps of the scarps of the central Brazilian shield. Former inhabitants of the flooded areas have been resettled in six newly created communities in the southern part of the lake region (Fig. 1). The proximity to the urban agglomeration of Cuiabá and Várzea Grande, with more than 700,000 inhabitants, has led to significant land transformation processes in the lake surroundings, with intense recreational activities, particularly on weekends, of a highly mobile population.

### Field work and data organization

156 adult vector collection campaigns were conducted at a total of 22 sites between May 2000 and September 2002. Three to five persons conducted sampling of adults during four-hour periods, elaborating a data set of human-biting rate (HBR) that followed human-biting collection techniques per WHO protocol [34]. Malaria transmission risk to researchers during entomological field survey can be considered low. During the sampling period no malaria case was reported for the study area.

At each site, captures were conducted with a mouth suction aspirator at four sampling points, in intra-, per- and extra-domicile situations and in four consecutive hourly periods, initiating first collection period at the start of sundown. Captured specimens were placed in vials containing 70% ethanol for later identification. Air temperature and relative humidity were measured during each hourly sampling period (Table 1). Lunar phase was registered for each sampling date.

**Table 1:** Non-spatial explanatory data sets evaluated in logistic regression models.

Explanatory variable	Description	Method	Scale	Number of classes
Season	Season	Wet: Nov.-April/Dry: May-Oct.	Nominal	2
Temperature	Air temperature	Digital thermometer	Interval	continuous
Humidity	Relative humidity	Digital humidity indicator	Ratio	continuous
Moon	Lunar phase	Field observation	Nominal	4

Maximum hourly biting-rates of one of the four sampling periods were used as the dependent variable.

All entomologic data sets, including geographical coordinates of sites and environmental conditions (temperature, relative humidity, lunar phase, wind speed) were joined in a database, developed under an Access/VB environment (Microsoft Inc.), which includes modules for the calculation of human biting rates differentiated for species.

### Spatial data sets and its processing

**Figure 2:**
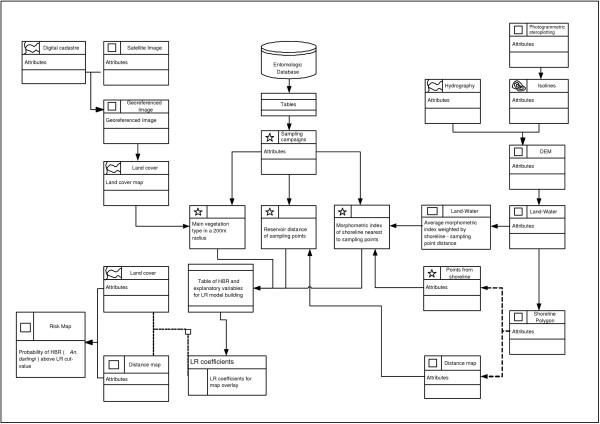
Data processing for habitat suitability mapping of *An. darlingi *in the APM Manso region. Data layers with no significant relationships with human bite rates are not considered.

Figure 2 gives an overview of the data processing sequence for *An. darlingi *habitat mapping in the Manso hydropower plant region. Geographic coordinates of sampling sites were determined by using a Garmin XL12 (Garmin Ltd., Olathe, Kansas) global positioning system (GPS) and exported into the Access 2000 (Microsoft) database of entomologic data and environmental field observations, which had been linked to an ArcView 3.2 GIS environment (ESRI, Redlands, CA).

All Remote Sensing and GIS data sets were georeferenced according to digital cadastre, available from FURNAS in a 1:25.000 scale UTM projection. Five spatial data layers were elaborated to examine its exploratory power of vector presence prediction (Table 2). Land cover was classified by Multispectral Landsat ETM imagery from July 2, 2000 (WRS 226/70). All six ETM bands with a 30 m spatial resolution (1–5, 7) were classified, applying the maximum likelihood algorithm implemented in the SPRING 4.2 GIS software (INPE, São José dos Campos, Brazil). As no high-resolution imagery was available at reasonable pricing for the evaluated period of field campaigns (2000–01), we had to base land cover mapping for habitat characterization exclusively on Landsat ETM imagery. A total of 57 ground truth sites were visited during field campaigns in 2000 and 2001, in a stratified sampling approach in order to obtain class samples from the complete extent of the study area. As land cover units are generally extent, average size of ground truth sites was about 54 ha. The classification algorithm was trained by half of the sites, whereas the other half was used for cross-validation. To parameterize land cover of vector sampling points, we determined the predominant cover in a 200 m radius, applying a majority filter with a 13 × 13 matrix. Land cover class was then numerical coded according to expected positive influence on vector densities (Pasture/farming: 1, Savannah: 2, Forest: 3).

**Table 2:** Spatial explanatory data sets evaluated in logistic regression models.

Explanatory variable	Description	Method	Data scale	layers
Slope	Slope	DEM analysis	ratio	1 layer
Aspect	Aspect	DEM analysis	ratio	1 layer
Land Cover	Vegetation/land use	Supervised classification of ETM data	nominal	1 layer, 3 classes
Distance	Distance of sampling point from water line at sampling date	DEM simulation, spatial queries	ratio	21 layers for each field sampling date
Shoreline index	Reservoir margin shape: relation of water and soil pixels	DEM simulation, convolution filter, overlay, spatial query	ratio	21 layers for each field sampling date

Digital elevation model was interpolated using plani-altimetric information from the 1:25.000 topographic map of the reservoir area applying the Topogrid algorithm [35] implemented in ArcInfo 7.2.1 (ESRI, Redlands, CA). Isolines with 1 m vertical resolution were combined with digitized river network as elaborated by FURNAS through photogrammetric stereoplotting before lake filling.

Slope and aspect layers were created applying standard GIS routines. A convolution filtering procedure with a 13 × 13 window was then applied to obtain averages weighted according to distance to vector sampling points in the 200 m radius.

Reservoir extension was modeled for each entomologic sampling date using water-level data monitored at the hydropower dam. Spatial querying was then applied to derive the site-reservoir distances during each entomologic sampling campaign.

A shape index of reservoir shoreline was developed based on the hypothesis that lake bays favor vector reproduction, as they are more protected from wind and waves than peninsulas. Shorelines simulated for each sampling data were segmented in 30 m arcs. Then a shape index for the arc nearest to each sampling site was determined by executing a sequence of spatial analysis procedures (Fig. 2). First, the land use layer was recoded into two classes: land and water. Through a region growing segmentation procedure, vector layers of shorelines at each entomologic sampling date were simulated. A spatial convolution filter with a 9 × 9 window, operating on the land-water layer was then applied to count and attribute number of water pixels in the neighborhood of each pixel. Overlay procedures were then applied to extract a land/water ratio for each sampling date. A distance map describing the distance of each shoreline to the next sampling site was then modeled. That way, shoreline pixels of a peninsula margin have ratios under 0.5, while shoreline pixels in bays have ratios above 0.5. Flight range estimates of *An. darlingi *vary between 2 and 7 km [2]. The comprehensive study of Keiser et al. [25] assumed a range of 2 km to estimate populations under malaria risk in dam influence areas. Considering the minimum water level of the reservoir, 19 of the 22 sampling points were located at a maximum distance of 2 km from the reservoir margin. For these points, a 2 km buffer was created and averaged shoreline indexes, weighted by the distance between shoreline elements and the sampling point. For the two remaining points (distance at minimum water level between 2 and 2.6 km respectively), we averaged weighted shoreline indexes in a radius of the minimum distance plus 10% (2.2 and 2.9 km respectively).

### Spatial model building by logistic regression

Logistic regression is a modeling technique for describing the relationship between a response variable and one or more explanatory variables, where the response variable follows a binomial distribution. To model the probability *p *of occurrence of a binary or dichotomous outcome, linear combination of the descriptor variables is taken, whose results are transformed to lie between 0 and 1. For analysis, the dependent variable, in our case the Human Bite Rate (HBR), has to be recoded according to a cut-off value, which determines the number of true positives, true negatives, false positives, and false negatives. The logistic regression model can be written as:

ln [p/(1-p)] = a + BX + e   (1)

where:

p: probability that the event Y occurs

ln [p/(1-p)]: log odds ratio, or "logit"

a: coefficient on the constant term,

B: coefficient(s) on the independent variable(s),

X: independent variable(s), and

e: error term.

The discrimination capacity of logistic regression models can be measured by cross-classifying observations and predictions in a two-by-two table, and calculating indices of classification performance [36] such as the Cox & Snell R Square. To overcome the essentially arbitrary choice of a cut-off value necessary in this approach, Receiver-operating characteristic (ROC) curves have been proposed for model validation. ROC evaluates the predictive accuracy over a range of threshold probabilities (cut-off) and can be graphically represented by plotting the false positive rate (1-specificity) against the true positive rate (sensitivity or 1 – the false negative rate) on the Y-axis. The accuracy of a logistic regression model test (i.e., the ability of the test to correctly classify cases with a certain condition and cases without the condition) can than be measured by the area under the ROC curve. An area of 1 represents a perfect test, while an area of 0.5 represents a worthless test. Statistical analysis were realized with SPSS, Rel. 10.0 (SPSS Inc., Chicago, IL)

## Results

### Human biting rates

**Figure 3:**
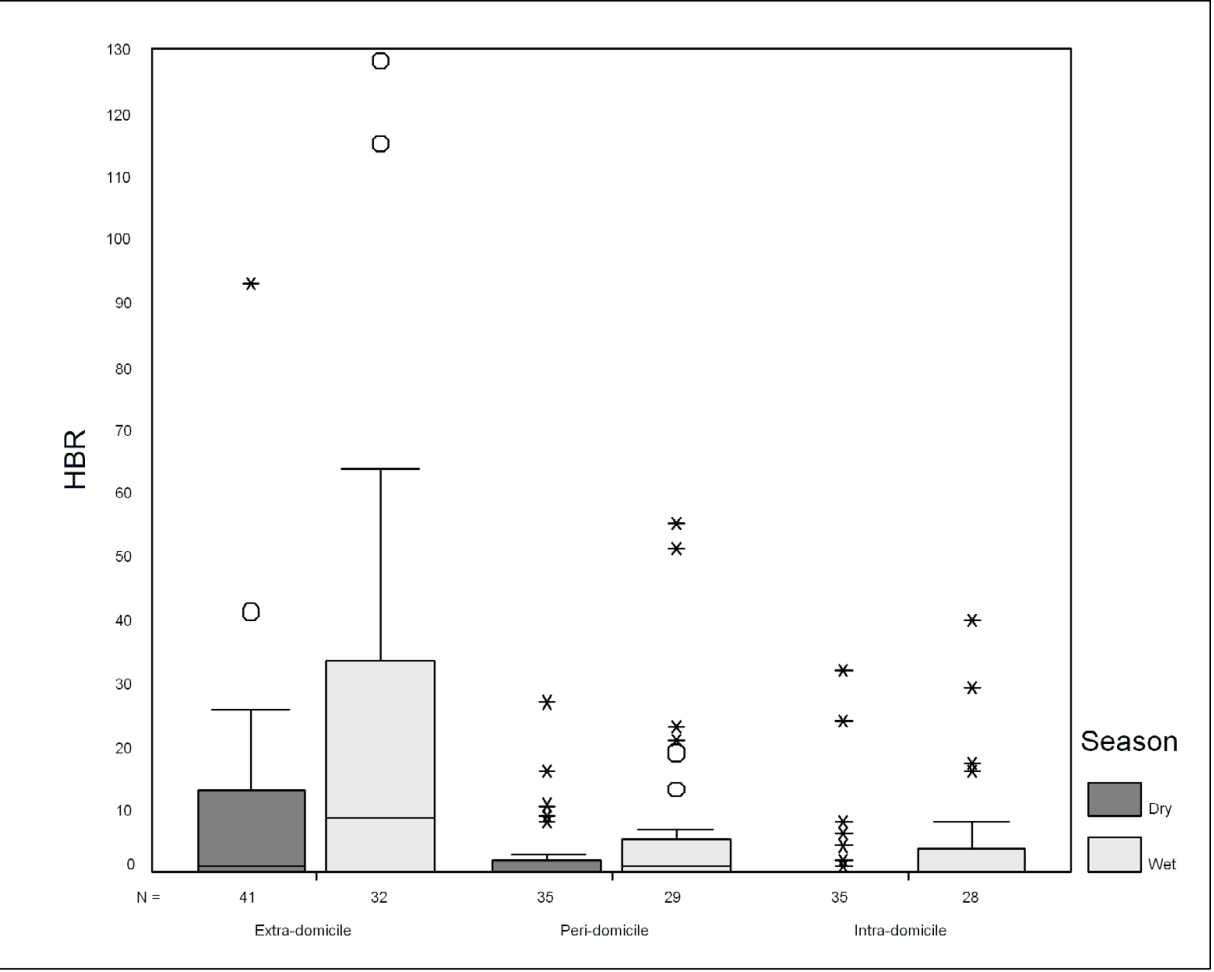
Human bite rates during the dry and wet seasons of the years 2000 through 2001 obtained from extra-, peri- and intra-domicile captures.

*An. darlingi *is widely described as an endophilic or endophagic [37]. In campaigns in and around housings of better quality, however, such as those realized in the residential areas of the hydroelectric stations of Samuel, Balbina, and Tucurui, only 0.6 % of *An. darlingi *specimens had been captured in the interior of houses. As shown in Fig. 3, maximum HBR from extra-domicile captures were much higher that those of peri-domicile and intra-domicile observations. Joining captures from both seasons, a paired sample Wilcoxon test results in highly significant differences (p < 0.01).

Following the argumentation of Charlwood [37], we suppose that these results could be a function of housing quality: all resettled families have obtained newly constructed houses. As we focus in the present study on the influence of environmental factors on habitat suitability we only considered extra-domicile samples for LR model building (n: 73). All evaluated sampling points, nevertheless, were inside *An. darlingi *flight ranges of rural settlement housing or tourist infrastructure (distance equal or less than 2 km).

Various studies have identified the relation between climatic conditions such as precipitation and temperature and human-biting rates of malaria vectors [27,36]. Figure 3 shows a comparison of human biting rates (HBR) of the 22 sampling points obtained from intra-, peri- and extra-domicile captures during two dry (March of 2000 – October 2000 and March of 2001 – October 2001) and two wet seasons (November of 2000 – April 2001 and November of 2001 – April 2002).

Exploratory analysis indicates slightly higher HBR during the wet season (Fig. 3), which could be expected due to an increase of reproduction habitats and higher water temperatures [38]. A maximum HBR value of 128 was obtained during a wet season campaign at the end of March 2000 near the reservoir. Captures of more than 10 specimens, however, were obtained in distances of more than 1.6 km from the reservoir, too. The median of HBR is higher for extra-domicile captures during the wet season (8.60) than for those of the dry season (1.25). A Wilcoxon test for paired samples, however, does not result in significant differences in vector incidences between the two periods (p = 0.328). Nevertheless, all meteorological explanatory data sets were tested in multiple LR model building to evaluate if spatial habitat simulations must be done for different meteorological-climatic conditions (see Tab. 4).

### Spatial data processing

**Figure 4:**
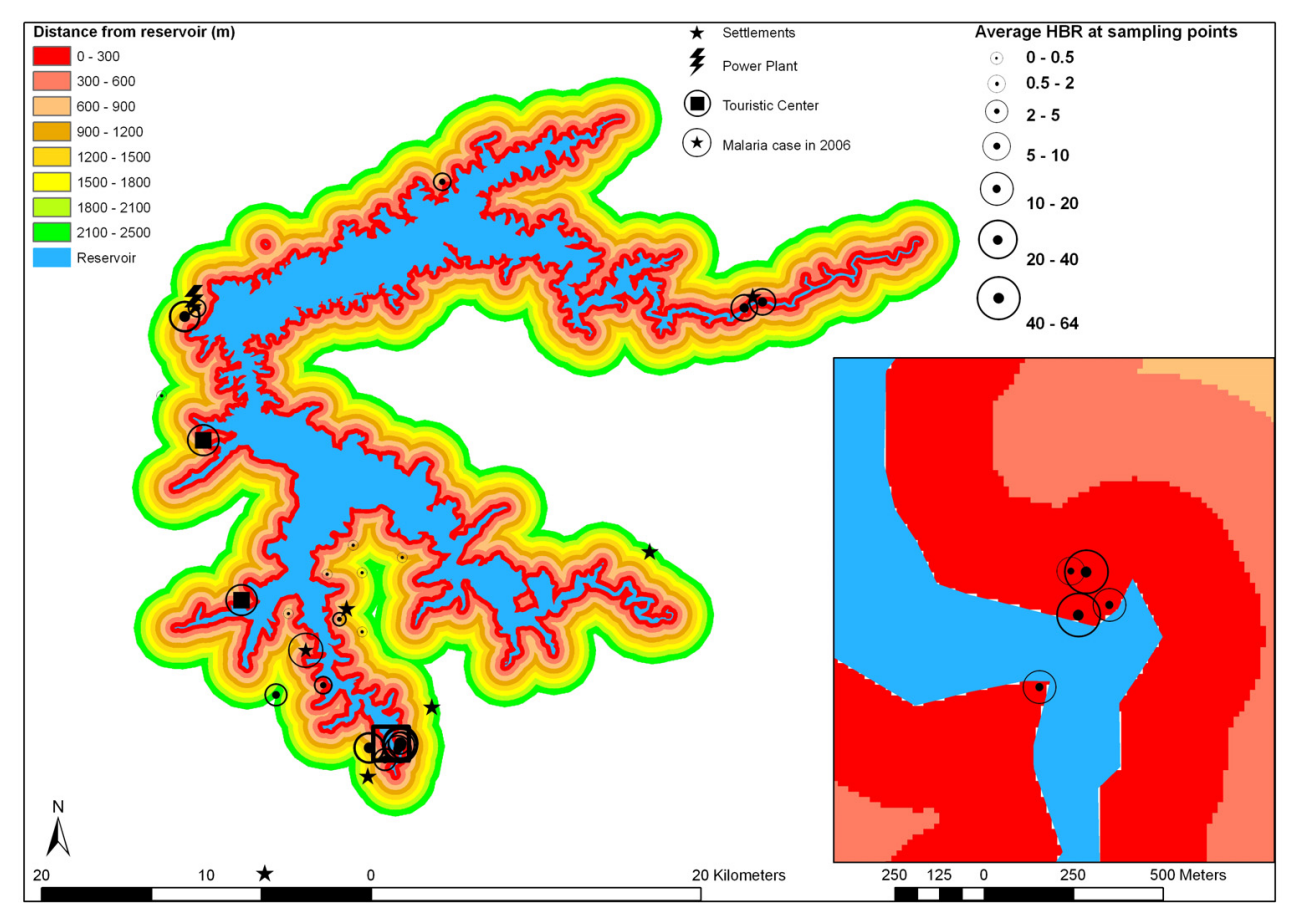
Distance map for the average high water level of the reservoir at 278.5 m NN.

Land cover classification was initially stratified in forest, savannah, cattle farming, crop farming/open soil and water. Only one small crop farming area was observed during field work. As this test site showed spectral signatures similar to recently reformed pastures, and as this land use is supposed to occupy less than 1 % of the reservoir influence area, pasture and crop farming areas were joined in one class in the final thematic layer. Proximity of sample points to waterbodies is already represented by the reservoir distance layer (Fig. 4). To minimize colinearity between explanatory data layers, percentages of water pixels in the 200 m radius around entomological sampling points were omitted.

**Figure 5:**
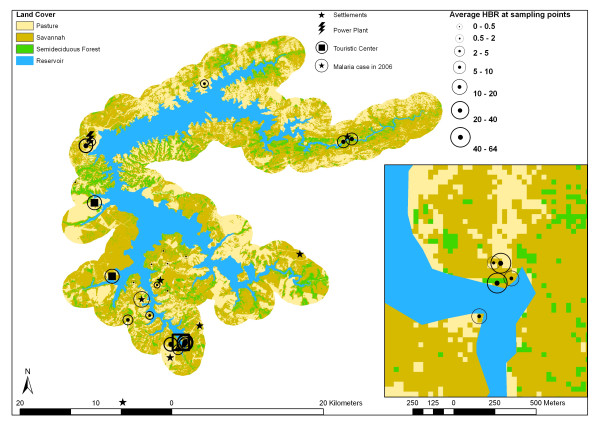
Land cover classification from Landsat ETM imagery with entomologic sampling points.

Table 3 presents the error matrix obtained from the Landsat ETM imagery classification for the three land cover classes used as descriptors in LR models. The result of supervised land cover classification is shown in figure 5, which is overlaid with scaled circles representing average HBR during the sampling period for the years 2000 through 2002.

**Table 3:** Error matrix of supervised Landsat ETM imagery classification (pixel counts of validation sites).

	*Reference data*			
	
*Classified data*	Pasture/Crop farming	Savannah	Forest	**User's accuracy**
Pasture/Crop farming	11257	1245	44	**89.73**
Savannah	2980	16075	502	**82.20**
Forest	40	673	1741	**70.97**

**Producer's accuracy**	**78.85**	**89.34**	**76.15**	**84.13**

**Table 4:** Coefficients (B) of multiple logistic regression (*"forward stepwise"*), applied for spatial modeling of *Anopheles darlingi *habitat suitability (HBR > 4).

		**B**	**S.E.**	**Wald**	**Sig.**	**Exp(B)**
Step 1	Season	.522	.627	.693	.405	1.686
	Slope	-.126	.146	.747	.388	.881
	Aspect	-.006	.003	4.413	.036	.994
	Moon	.117	.265	.197	.657	1.124
	Temperature	-.010	.060	.027	.869	.990
	Humidity	.004	.022	.028	.868	1.004
	Land Cover	.721	.747	.932	.334	2.057
	Shoreline	2.240	2.803	.639	.424	9.395
	Distance	-.356	.197	3.266	.071	.701

Step 2	Season	.512	.624	.674	.412	1.669
	Slope	-.129	.146	.786	.375	.879
	Aspect	-.006	.003	4.528	.033	.994
	Moon	.094	.226	.175	.676	1.099
	Humidity	.003	.021	.016	.898	1.003
	Land Cover	.720	.749	.924	.337	2.054
	Shoreline	2.074	2.599	.637	.425	7.955
	Distance	-.365	.191	3.641	.056	.694

Step 3	Season	.555	.526	1.113	.292	1.742
	Slope	-.126	.142	.780	.377	.882
	Aspect	-.005	.003	4.569	.033	.995
	Moon	.099	.224	.194	.660	1.104
	Land Cover	.748	.714	1.096	.295	2.113
	Shoreline	2.092	2.593	.651	.420	8.098
	Distance	-.357	.181	3.888	.049	.700

Step 4	Season	.611	.509	1.445	.229	1.843
	Slope	-.108	.134	.656	.418	.897
	Aspect	-.005	.003	4.410	.036	.995
	Land Cover	.758	.697	1.183	.277	2.134
	Shoreline	1.929	2.533	.580	.446	6.881
	Distance	-.332	.168	3.923	.048	.717

Step 5	Season	.704	.497	2.010	.156	2.022
	Slope	-.080	.128	.392	.531	.923
	Aspect	-.005	.002	4.114	.043	.995
	Land Cover	.994	.636	2.439	.118	2.701
	Distance	-.281	.153	3.375	.066	.755

Step 6	Season	.628	.478	1.728	.189	1.874
	Aspect	-.005	.002	3.976	.046	.995
	Land Cover	.673	.361	3.465	.063	1.960
	Distance	-.209	.095	4.781	.029	.812

Step 7	Aspect	-.004	.002	3.200	.074	.996
	Land Cover	.965	.294	10.792	.001	2.625
	Distance	-.163	.086	3.583	.058	.850

**Step 8**	**Land Cover**	**.693**	**.251**	**7.629**	**.006**	**1.999**
	**Distance**	**-.222**	**.080**	**7.642**	**.006**	**.801**

Overall accuracy for the three land cover classes used as predictors in LR model building was determined to be 84.1. Producer's and User's accuracies are similarly high for Pasture and Savannah formations, whereas forest classification is less accurate. Denser savannah woodlands ("Cerradão"), build ecotones with the semi-deciduous forests, causing higher rates of misclassifications between both classes. Thematic map (Fig. 5) shows Savannah and Pastures as the predominant land cover. Semi-deciduous forests, the vegetation formation where the highest HBR were obtained, covers the dissected steep ramps of the Central Brazilian shield and accompany the stream valleys.

**Figure 6:**
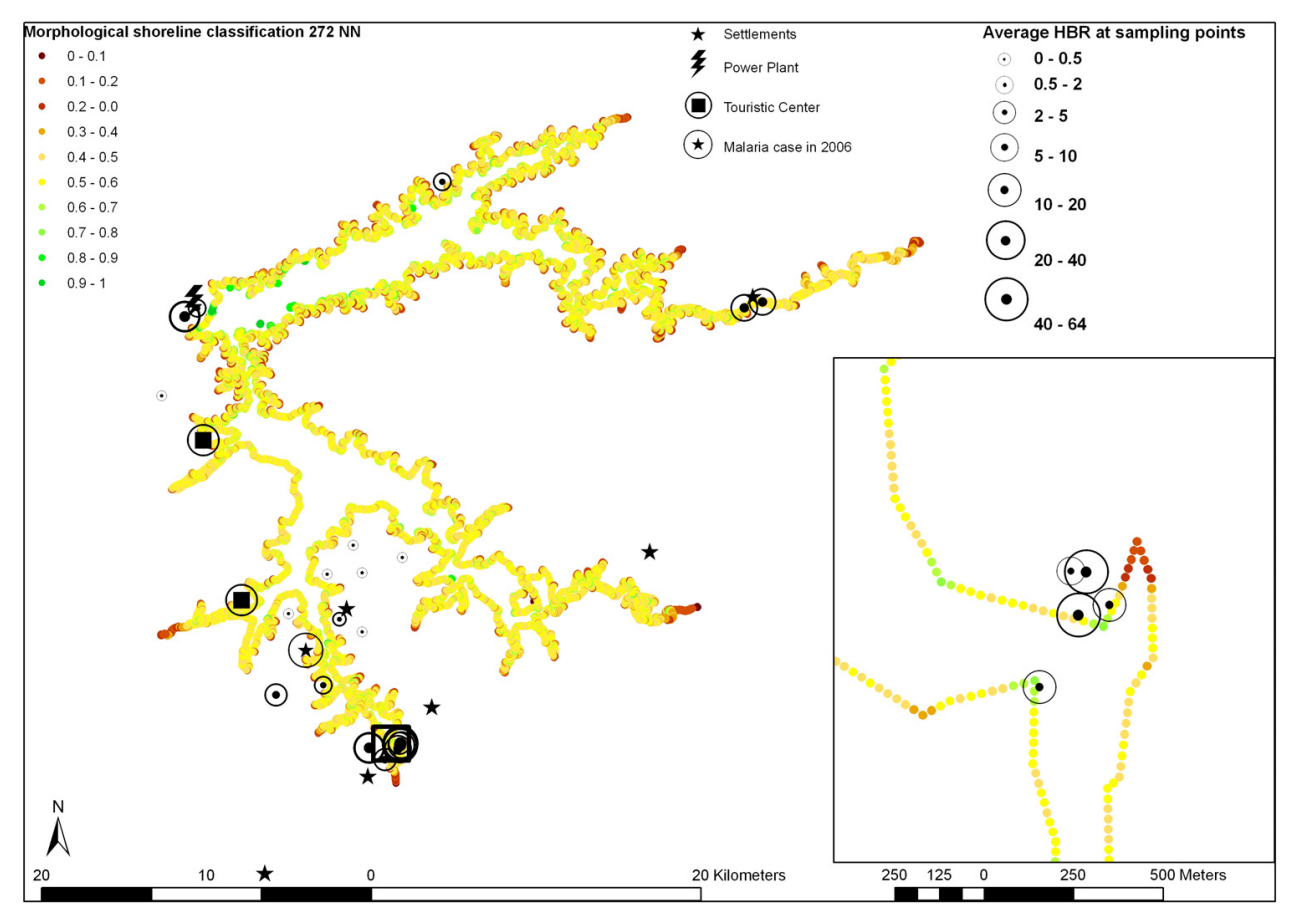
shows a detail of morphological shoreline classification.

Figures 4 and 6 expose the distance map and shoreline shape classification for the entire reservoir area as well as detailed subsets. Figures are overlaid with scaled circles representing average HBR during the sampling period for the years 2000 through 2002.

From the 73 extra-domicile sampling campaigns, 31 were carried out at a distance between 0 a 200 m of reservoir shoreline, 12 at a distance between 200 and 500 m, 25 between 500 and 1,000 m and 5 at a distance greater than 1 km (Fig. 4).

**Figure 7:**
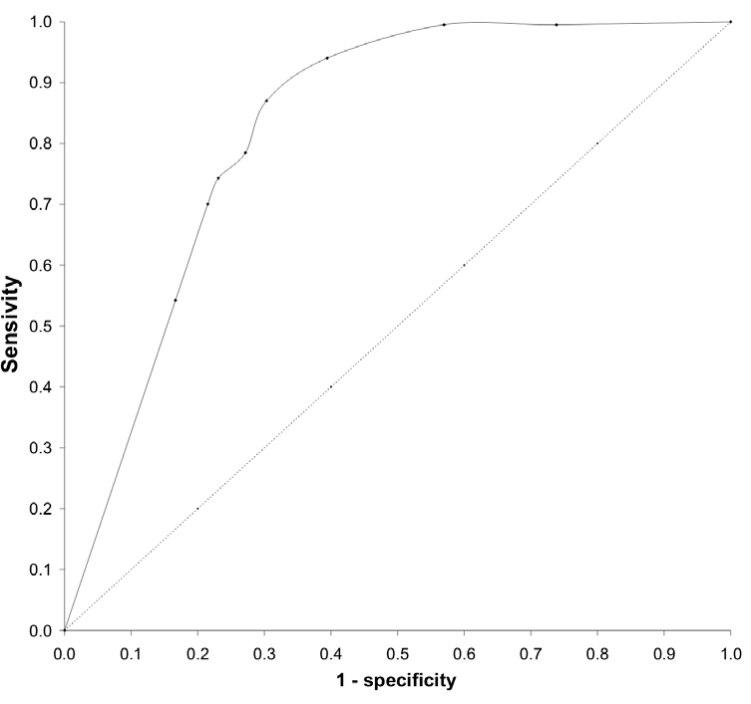
ROC curve of HBR cut-off values of *Anopheles darlingi*.

Figure 7 represents the ROC curve with sensitivity and specificity obtained for LR models with cut-off values of 0.5, 1, 2, 4, 6, 10, 20, and 50. The area under the ROC was found to be 82.51%, considered to be a satisfactory global predictive power [39]. Best equilibrium between sensivity and specificity was obtained for a cut-off value of 6. For final model building, however, we opted for a cut-off value of 4, since positive cases are poorly predicted for cut-off values of 6 and 10.

**Figure 8:**
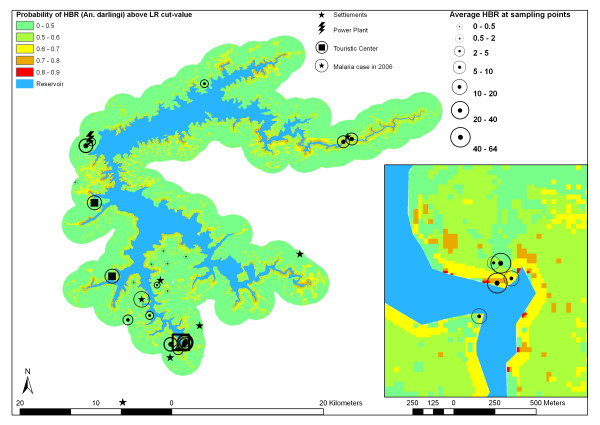
Probability of HBR (*Anopheles darlingi*) above LR cut-value.

Table 4 summarizes LR outcomes for a cut-off value of 4. Spatial model of habitat suitability in the Manso hydropower plant influence area is shown in Figure 8. Shorter distances to the reservoir border are positively related to mosquito presence. Semi-deciduous forests (coded as three for LR) are the best habitats for *An. darlingi*, followed by savannah.

## Discussion

Although *An. darlingi *is the most important malaria vector in Brazil, knowledge about its habitats is limited, particularly on a local scale and in the influence area of hydropower plant reservoirs.

Various studies have reported the importance of climatic factors on vector incidence and malaria prevalence in semi-humid tropical climates; increased rain and temperatures, for example, can have positive effects on vector breeding and development rates [21]. Our results indicate that the availability of a permanent reproduction habitat seems to equilibrate seasonal differences in vector incidence generally observed in humid and semi-humid tropical climates [22]. Slightly higher vector densities observed at collections during the rainy season (November through April) were not found to be significantly superior to those of the dry season (p < 0.05). Similarly, Guimareas et al. [26] in their study on the Itaipu power plant reservoir mentioned that *Anopheline *incidence did not increase during the summer rainy season. Contrary to our findings, *An. darlangi *incidences in the surroundings of the Serra da Mesa power plant in the Cerrado savannah region of Goias state was found to be higher during the rainy season [23].

Multispectral Landsat ETM imagery classification and DEM-based GIS methods were applied to local scale prediction of vector presence probabilities. As climatic factors showed no significant influence on vector presence, only a unique logistic regression model was developed.

First, higher incidences are associated with spatial proximity to reservoir and semi-deciduous forests followed by Cerrado woodland. Vector absence was associated with open vegetation formations such as natural grasslands, pastures and agricultural areas. As in our study, Guimaraes et al. [23] noted higher incidences of *An. darlingi *for locations covered with denser savannah formations ("Cerrado", "Cerradão"). None of their sampling sites, however, was covered by semi-deciduous forest, which showed the highest incidences in our study. It is important to mention that vegetation units in the hilly terrain of the Manso power plant region are highly fragmented, reflecting variability in geoecological conditions as well as anthropic transformations. Forest formations in the study area are spatially limited to corridors of straight river valleys along the outlets of inflowing streams. Semi-deciduous forests as well as *Cerrado *woodlands and savannahs are reminiscent of patches interlaced by managed pastures. None of the sampling points was located in a continuous singular vegetation unit whose area would exceed the flight ranges of *An. darlingi*. Therefore, we see no contradiction to studies that have reported an increase of *An. darlingi *incidence in Amazon regions suffering recent deforestation [20,26]. As rural populations predominantly settle near stream valleys covered by forest, vector presence may be additionally favored by the availability of feeding sources.

Maximum flight range of *An. darlingi *is estimated to vary between 2 km and 7 km [2]. As pointed out by Charlwood & Wilkes [40], *An. darlingi *may follow edges of the forest margin following road systems. As the implantation of resettlements and tourist infrastructure has opened potential flight paths, we believe that mosquitoes may reach areas in a distance of at least 2.5 km from the supposed breeding habitats in the hydropower reservoir. This thesis seems to be confirmed by some HBR above 10 in a distance more than 1.6 km from the shoreline. In reservoirs such as that of the Manso power plant, which experience extensive water level variations as a function of operation schemas for power generation and seasonal hydrological stream regimes, distance estimates based on DEM analysis should be done for date specific water levels. Maximum water level amplitude of about 10 m can result in horizontal alterations of shoreline distances of more than 200 m. Vector presence and reservoir proximity had significant positive correlation in an univariate as well as in the multivariate LR model (p < 0.05). Since preferred breeding habitats of *An. darlingi *are large pools of stagnant water and slow-moving streams [23], we developed and tested a shoreline morphographic index based on DEM analysis. In an univariate LR model, vector absence was significantly related to peninsula situations (index > 0.5), when vector incidence was elevated at sampling points near embayments, protected from wave action. In the multivariate LR model, however, shoreline index was rejected as an explanatory variable in a 0.05 confidence interval. Results of nonparametric correlation analysis suggest that this is due to correlation of shoreline index with vegetation types. Sampling points with high vector densities near reservoir bays – in many cases flooded stream valleys – are mainly covered by dense wooden vegetation formations.

## Conclusion

According to the presented results, we conclude in the framework of formulated hypothesis: the incidence of *An. darlingi *in the surroundings of the Manso hydropower plant in the Central Brazilian *Cerrado *region is highest near the reservoir and inside semi-deciduous forest. Savannah scrub- and woodlands are more suitable habitats than pastures or cropland.

Only slight seasonal differences among HBR were observed, indicating that the increase of mosquito presence in the wet season as reported for other sub-humid tropical regions is balanced by the presence of an artificial reproduction habitat.

Remote Sensing and GIS techniques such as digital land cover classification and DEM based buffering were found to contribute to habitat characterization and mapping. GIS-based morphographic classification, still little explored for habitat suitability simulation, was found to be a promising technique that should be included in future studies of *An. darlingi *habitats.

## Authors' contributions

PZ and ESS conceived the study design, performed statistical and spatial analysis and drafted the manuscript. AR and RM carried out the entomologic survey and participated in data analysis. MS participated in study design and was responsible for project coordination. All authors participated in the elaboration of final manuscript.

## References

[B1] Lourenço-de-Oliveira R (1995). Qual a importância da hematofagia extradomiciliar do Anopheles darlingi na Amazônia?. Rev Patol Trop.

[B2] Charlwood J, Alecrim WA (1989). Capture-recapture studies with the South American Malaria vector Anopheles darlingi, Root. Annals of Tropical Medicine & Parasitology.

[B3] Roberts DR, Laughlin LL, Paul H, Llewellyn JL (1997). DDT, Global Strategies, and a Malaria Control Crisis in South America. Emerging Infectious Diseases.

[B4] WHO/RBM (2005). World Malaria Report. http://www.rbm.who.int/wmr2005/.

[B5] Gurgel HC (2003). A Utilização das Geotecnologias em Estudos Epidemiologicos: O Exemplo da relação entre a Malária e o NDVI em Roraima. Annals XI SBSR, Belo Horizonte, Brazil, 05–10 april INPE.

[B6] (2004). Agência Nacional de Energia Elétrica. Informações do Setor Elétrico.

[B7] Consolim J, Luz E, Pellegrini NJM, Torres PB (1991). O Anopheles (Nyssorhynchus) darlingi Root, 1926 e a Malária no Lago de Itaipu, Estado do Paraná, Brasil: Uma revisão de dados (Díptera, Culicidae). Technology and Biology Files.

[B8] Clarke KC, Mc Lafferty SL, Tempalski BJ (1996). On epidemiology and geographic information systems: a review and discussion of future directions. Emerg Infect Dis.

[B9] Beck LR, Bradley ML, Byron LW (2000). Remote Sensing and Human Health: New Sensors and New Opportunities. Emerg Infect Dis.

[B10] Thomson MC, Connor SJ, Milligan PJM, Flasse SP (1996). The ecology of malaria as seen from Earth-observation satellites. Ann Trop Med Parasitol.

[B11] Rogers DJ, Randolph SE, Snow RW, Hay SI (2002). Satellite imagery in the study and forecast of malaria. Nature.

[B12] Pope KO, Rejmánková E, Savage HM, Arredondo-Jimenez JI, Rodríguez MH, Roberts DR (1993). Remote sensing of tropical wetlands for malaria control in Chiapas, Mexico. Ecological Applications.

[B13] Beck LR, Rodriguez MH, Dister SW, Rodriguez AD, Rejmankova E, Ulloa A, Meza RA, Roberts DR, Michael AS, Washino RK, Hacker C, Legters LJ (1994). Remote sensing as a landscape epidemiologic tool to identify villages at high risk for malaria transmission. Am J Trop Med Hyg.

[B14] Roberts DR, Paris JF, Manguin S, Harbach RE, Woodruff R, Rejmankova E, Polanco J, Wullschleger B, Legters LJ (1996). Predictions of malaria vector distribution in Belize based on multispectral satellite data. Am J Trop Med Hyg.

[B15] Mushinzimana E, Munga S, Minakawa N, Li L, Feng C, Bian L, Kitron U, Schmidt C, Beck L, Zhou G, Githeko AK, Yan G (2006). Landscape determinants and remote sensing of anopheline mosquito larval habitats in the western Kenya highlands. Malaria Journal.

[B16] Minakawa N, Munga S, Atieli FK, Mushinzimana E, Zhou G, Githeko AK, Yan G (2005). Spatial distribution of anopheline larval habitats in western Kenya highlands: Effects of land cover types and topography. Am J Trop Med Hyg.

[B17] Vasconcelos CH, Novo EMLM, Donalisio MR (2006). Use of remote sensing to study the influence of environmental changes on malaria distribution in the Brazilian Amazon. Cadernos de Saúde Pública.

[B18] Takken W, Vilarinhos PTR, Schneider P, Santos F, Bogers RJ, Martens P, Takken W (2005). Effects of environmental change on malaria in the Amazon region of Brazil. Environmental Change and Malaria Risk Global and Local Implications.

[B19] Deane LM (1986). Malaria vectors in Brazil. Memorias do Instituto Oswaldo Cruz.

[B20] Vittor AY, Gilman RH, Tielsch J, Glass G, Shields T, Lozano WS, Pinedo-Cancino V, Patz JA (2006). The effect of deforestation on the human-biting rate of Anopheles darlingi, the primary vector of Falciparum malaria in the Peruvian Amazon. Am J Trop Med Hyg.

[B21] Castro MC, Monte-Mor RL, Sawyer DO, Singer BH (2006). Malaria risk on the Amazon frontier. Proc Natl Acad Sci USA.

[B22] Singer BH, Castro MC (2001). Agricultural Colonization and Malaria on the Amazon Frontier. Annals of the New York Academy of Sciences.

[B23] Guimaraes AE, Gentile C, Alencar J, Lopes CM, de Mello RP (2004). Ecology of Anopheline (Diptera, Culicidae), malaria vectors around the Serra da Mesa Reservoir, State of Goias, Brazil. 1 – Frequency and climatic factors. Cad Saude Publica.

[B24] Hakre S, Masuoka P, Vanzie E, Roberts DR (2004). Spatial correlations of mapped malaria rates with environmental factors in Belize, Central America. International Journal of Health Geographics.

[B25] Keiser J, Caldas de Castro M, Maltese MF, Bos R, Tanner M, Singer BH, Utzinger J (2005). Effect Of Irrigation And Large Dams On The Burden Of Malaria On A Global And Regional Scale. Am J Trop Med Hyg.

[B26] Guimarães AE, Mello RP, Lopes CM, Alencar J, Gentile C (1997). Prevalência de Anofelinos (Diptera: Culicidae) no Crepúsculo Vespertino em Áreas da Usina Hidrelétrica de Itaipu, no Município de Guaíra, Estado do Paraná, Brasil. Mem Inst Oswaldo Cruz.

[B27] Tadei WP, Thatcher BD, Santos JM, Scarpassa VM, Rodrigues IB, Rafael MS (1998). Ecologic observations on anopheline vectors of malaria in the Brazilian Amazon. Am J Trop Med Hyg.

[B28] Chambo OY (2002). Preventive Actions against Malaria-A Study of Factors Influencing the Use and Re-Impregnation of Bed Nets in Highland Villages in Tanzania. Master thesis.

[B29] Singh N, Mehra RK, Sharma VP (1999). Malaria and the Narmada-river development in India: a case study of the Bargi dam. Ann Trop Med Parasitol.

[B30] Hoffmann EJ, Miller JR (2003). Reassessment of the Role and Utility of Wind in Suppression of Mosquito (Diptera: Culicidae) Host Finding: Stimulus Dilution Supported Over Flight Limitation. Journal of Medical Entomology.

[B31] Voorham J (2002). Intra-population plasticity of Anopheles darlingi's (Diptera, Culicidae) biting activity patterns in the state of Amapá, Brazil. Rev Saúde Pública.

[B32] Duarte AMRDC, Porto MAL, Curado I, Malafronte RS, Hoffmann EHE, Oliveira SGD, Silva AMJD, Kloetzel JK, Gomes ADC (2006). Widespread occurrence of antibodies against circumsporozoite protein and against blood forms of Plasmodium vivax, P. falciparum and P. malariae in Brazilian wild monkeys. J med primatol.

[B33] Ratter JA, Bridgewater S, Atkinson R, Ribeiro JF (1996). Analysis of the floristic composition of the Brazilian Cerrado vegetation: II. Comparison of the woody vegetation of 98 areas. Edinburgh Journal of Botanics.

[B34] World Health Organization (1975). Manual on practical entomology in malaria. Part II. Methods and techniques. Ginebra WHO (WHO Offset Publication 13).

[B35] Hutchinson MF (1989). A new procedure for gridding elevation and stream line data with automatic removal of spurious pits. Journal of Hydrology.

[B36] Pearce J, Ferrier S (2000). Evaluating the predictive performance of habitat models developed using logistic regression. Ecological Modelling.

[B37] Charlwood JD (1996). Biological Variation in *Anopheles darlingi *Root. Mem Inst Oswaldo Cruz.

[B38] Freeman T, Bradley M (1996). Temperature is predictive of severe malaria years in Zimbabwe. Transactions of the Royal Society of Tropical Medicine and Hygiene.

[B39] Fortes BPMD, Valencia LIO, Ribeiro SV, Medronho RA (2004). Geostatistical modeling of Ascaris lumbricoides infection. Cad Saúde Pública.

[B40] Charlwood JD, Wilkes TJ (1981). Observations on the biting activity of Anopheles triannulatus bachmanni from the Mato Grosso, Brazil. Acta Amazonica.

